# Detection of nirsevimab-resistant RSV-B variants in children carrying the F:N201T substitution through genomic surveillance, Spain, 2025/26 season

**DOI:** 10.2807/1560-7917.ES.2026.31.35.2600690

**Published:** 2026-09-03

**Authors:** María Iglesias-Caballero, Jose A Infantes, Albert Campoy, Sara Camarero-Serrano, Silvia Moreno, Olga Cano, Germán Vallejo-Palma, Carmen González-Velasco, Cristina Gaona, Pedro Camacho- Martinez, Laura Merino-Diaz, Sonia Vázquez-Morón, Francisco Pozo, Jose A Lepe, Vicente Mas, Inmaculada Casas

**Affiliations:** 1 Laboratory of Reference and Research in Respiratory Viruses, National Centre for Microbiology, Instituto de Salud Carlos III, Majadahonda, Spain; 2 Clinical Unit of Microbiology, University Hospital of Badajoz, Extremadura, Spain; 3 Clinical Unit of Infectious Diseases, Microbiology and Parasitology, University Hospital Virgen del Rocío, Seville, Spain; 4 Institute of Biomedicine of Seville (IBiS), University Hospital Virgen del Rocío/CSIC/University of Seville, Seville, Spain; 5 CIBER de Epidemiología y Salud Pública (CIBERESP), ISCIII, Madrid, Spain

**Keywords:** respiratory syncytial virus, viral evolution, genomic surveillance, nirsevimab

## Abstract

During Spain’s 2025/26 RSV season, three children were infected with RSV-B viruses carrying the F protein N201T substitution. Two who received nirsevimab that season were hospitalised (one required intensive care); the third was treated previously. Recombinant expression studies confirmed reduced nirsevimab recognition. Genomic data indicated sporadic emergence across distinct RSV-B backgrounds rather than expansion of a single lineage. Findings highlight the importance of combining genomic surveillance with antigenic characterisation to detect and interpret variants with reduced susceptibility to antibody-based prevention.

During the 2025/26 respiratory syncytial virus (RSV) season, Spanish national genomic surveillance identified three children infected with RSV subtype B (RSV-B) viruses carrying the F protein N201T substitution in antigenic site Ø, the target of nirsevimab; two of the children were hospitalised following infection. Two had received nirsevimab during the current season, one of whom developed severe bronchiolitis requiring paediatric intensive care unit (PICU) admission and non-invasive ventilatory support. We investigated the clinical characteristics, antigenic phenotype and evolutionary context of these viruses to assess the public health relevance of this uncommon substitution.

## Clinical detection and case description

The three cases were identified through the Spanish SiVIRA laboratory surveillance network [1], a national surveillance system which monitors acute respiratory infections in primary care and hospitals. Two cases with viruses harbouring the F:N201T substitution were detected in Andalusia and one in Extremadura, among 951 RSV-B sequences identified nationally. The two children from Andalusia were born during the 2025/26 season, whereas the child from Extremadura was born before that season. Two of the three children were male, one was female, and two children for whom gestational age was available were born at term (Table 1).

**Table 1:** Clinical characteristics of three children infected with RSV-B carrying the F:N201T substitution, Spain, season 2025/26

Characteristics	Case 1	Case 2	Case 3
Virus name	hRSV/B/Spain/AN-PMC-00541/2025	hRSV/B/Spain/AN-PMC-00612/2026	hRSV/B/Spain/EX-BA 18805365/2026
Underlying conditions	Down syndrome; complete AV canal defect	None	None
Previous nirsevimab	Yes	Yes	Yes, season 2023/24
Days from 2025/26 nirsevimab to hospital admission	52–82 days^a^	99 days	N/A
Main symptoms	Fever, cough, rhinorrhoea	Fever, cough, rhinorrhoea	Cough, odynophagia, diarrhoea
Lowest SpO_2_ (on admission)	90%	92%	N/A
Oxygen therapy	Low-flow nasal oxygen (1.5 L/min)	Escalated to BiPAP	N/A
PICU admission	No	Yes	No
Mechanical ventilation	No	Non-invasive ventilation (BiPAP)	N/A
Chest imaging	Bilateral infiltrates	Bilateral infiltrates	N/A
Hospital length of stay	7 days	8 days (2 in PICU)	N/A
Outcome	Full recovery	Full recovery	Full recovery

AV: atrioventricular; BiPAP: bilevel positive airway pressure; N/A: not applicable; PICU: paediatric intensive care unit; RSV-B: respiratory syncytial virus subtype B; SpO_2_: peripheral oxygen saturation.

^a^ Estimated range; exact administration date unavailable.

Case 1, the patient infected with hRSV/B/Spain/AN-PMC-00541/2025, had Down syndrome and a complete atrioventricular canal defect. They had received nirsevimab before infection and were admitted to the hospital with fever, cough and rhinorrhoea. The lowest oxygen saturation was 90%, bilateral infiltrates were observed on chest imaging, and they required low-flow nasal oxygen. The patient recovered after a 7-day hospital stay.

Case 2 was infected with hRSV/B/Spain/AN-PMC-00612/2026, had no reported underlying conditions and had also received nirsevimab before infection. They developed bronchiolitis with fever, cough and rhinorrhoea and required escalation to bilevel positive airway pressure. They were admitted to the hospital for 8 days, including 2 days in the PICU, and recovered fully.

Case 3, infected with hRSV/B/Spain/EX-BA 18805365/2026, presented to the emergency department with cough, odynophagia and diarrhoea but did not require hospitalisation. They had received nirsevimab during the 2023/24 season, rather than during the season in which infection occurred. This case should therefore not be considered a current-season breakthrough infection, but it indicates that F:N201T-positive viruses may also be detected in the community without documented recent nirsevimab exposure.

## Genomic context of the F:N201T substitution

To investigate the evolutionary context of F:N201T, we searched all partial F-gene and whole genome RSV-B sequences available. Eight sequences carrying the substitution were identified (Table 2). The earliest detections were from Germany in season 2001/02, followed by the United States in season 2017/18 and South Africa in season 2024. Four sequences were from Spain: one detected in Valencia in season 2024/25 (hRSV/B/VC-GRAL-0030011/2025) and the three associated to Cases 1–3 of this study, submitted by the sequencing services of the participating hospitals. The four Spanish viruses belonged to lineages B.D.E.1, B.D.E.1.1 and B.D.E.1.2.

**Table 2:** RSV-B sequences carrying the F:N201T substitution available in public databases, globally, seasons 2001/02–2025/26 (n = 8)

Virus name	Spanish case	Geographic location	RSV season of sample collection	Lineage	GISAID ID
hRSV/B/Germany/un-RKI-2173/2002	N/A	Germany	2001/02	B	EPI_ISL_18891844
hRSV/B/Germany/BY-02–02173/2002	N/A	Germany	2001/02	B	EPI_ISL_18005951
hRSV/B/US/HI-AZL-09042944/2018	N/A	United States	2017/18	B.D.4.1.1	EPI_ISL_17218082
hRSV/B/South_Africa/NICD-R02620/2024	N/A	South Africa	2024^a^	B.D.4.1.1	EPI_ISL_19220841
hRSV/B/Spain/VC-GRAL-003011/2025	N/A	Spain	2024/25	B.D.E.1.1	EPI_ISL_20082441
hRSV/B/Spain/AN-PMC-00541/2025	Case 1	Spain	2025/26	B.D.E.1.2	EPI_ISL_20335071
hRSV/B/Spain/AN-PMC-00612/2026	Case 2	Spain	2025/26	B.D.E.1.2	EPI_ISL_20368155
hRSV/B/Spain/EX-BA_18805365/2026	Case 3	Spain	2025/26	B.D.E.1	EPI_ISL_20383287

N/A: not applicable; RSV-B: respiratory syncytial virus subtype B.

^a^ As South Africa is in the Southern Hemisphere, the winter respiratory season falls within a single calendar year.

Sequence data were obtained from the GISAID [2] and GenBank databases, including the three sequences corresponding to the Spanish cases described in this study, which were submitted by the sequencing services of the participating hospitals, as specified above. Seven of the eight F:N201T sequences were whole genome sequences (> 95% genome coverage) generated using Illumina or Nanopore sequencing platforms; EPI_ISL_17218082 covered only the F gene.

We next compared the amino acid substitution profiles of the four Spanish viruses relative to reference strain NC_001781.1 (Table 3). The viruses from Case 1 and Case 2 both detected in Andalusia showed highly similar genomic profiles, whereas the virus from Case 3 (Extremadura) and the Valencia virus displayed distinct combinations of substitutions. Both Andalusian viruses lacked the F protein Q209R substitution, which is otherwise highly prevalent among contemporary RSV-B viruses. No consistent genomic signature other than F:N201T was shared by all four Spanish viruses.

**Table 3:** Distinctive amino acid substitution profiles of RSV-B viruses carrying F:N201T, Spain, seasons 2024/25 and 2025/26 (n = 4)

RSV protein	hRSV/B/Spain/VC-GRAL-003011/2025	hRSV/B/Spain/AN-PMC-00541/2025 (Case 1)	hRSV/B/Spain/AN-PMC-00612/2026 (Case 2)	hRSV/B/Spain/EX-BA 18805365/2026 (Case 3)
NS1	K74E	None	None	None
NS2	None	D6N, N123K	D6N, N123K	E83K
P	D225N	P73S	P73S	None
SH	T49M	T49I	T49I	T49I
G	T107A, T118I, K258N, I271A	T107V, A116T, S249P, T256I, S267P, I271V, T275I	T107V, A116T, S249P, T256I, S267P, I271V, T275I	T107A, T118I, F168Y, K258N, S267P, I271A, T276I
F	Q209R, S389L,	F12I, K87R, S389P, S362F, I544V	F12I, K87R, S389P, I544V	R42K, Q209R, S389P
M2-1	N182S	N182S	N182S	None
L	Y86F, I185V, N590S	S169P, H177Y, I284M, E1714G, L1809F, L1813F	S169P, H177Y, I284M, E1714G, L1809F, L1813F	H177Y, V1941I, V1965I

RSV-B: respiratory syncytial virus subtype B.

For simplicity, only amino acid substitutions that distinguish the profiles of the four Spanish viruses are shown. Substitutions were defined relative to reference strain NC_001781.1, except for those within the 60-nt duplicated region of the G protein (aa 258–277), which were defined relative to contemporary RSV-B reference/ EPI_ISL_1653999, which contains the duplication. For viruses associated with the cases described in this study, the corresponding case number is indicated in parentheses after the virus name. ‘None’ indicates that no amino acid substitutions were identified for the corresponding protein.

Together with the detection of F:N201T across different countries, years and RSV-B lineages, these findings support recurrent emergence across distinct genetic backgrounds rather than expansion of a single lineage. Nevertheless, the similarity between the two Andalusian viruses suggests that local transmission cannot be excluded.

## Antigenic effect in contemporary RSV-B viruses

Because virus isolation from the clinical specimens was unsuccessful, we evaluated antibody binding using recombinant full-length prefusion F proteins from contemporary RSV-B viruses transiently expressed in HEK293-F cells as previously described [3]. Cells were harvested 48 h post-transfection, and relative binding of nirsevimab, clesrovimab and suptavumab was measured by flow cytometry and normalised to palivizumab binding.

The F protein derived from the contemporary virus associated with Case 2 (hRSV/B/Spain/AN-PMC-00612/2026) carrying the N201T substitution showed markedly reduced nirsevimab binding compared with F protein of the reference strain CH18537 (Figure). Analysis of the historical antigenic site Ø background in which N201T had previously been described reproduced the reduced-recognition phenotype. Conversely, reversion of threonine to asparagine at residue 201 partially restored nirsevimab binding, confirming a direct contribution of N201T despite the presence of additional contemporary substitutions surrounding antigenic site Ø.

**Figure:**
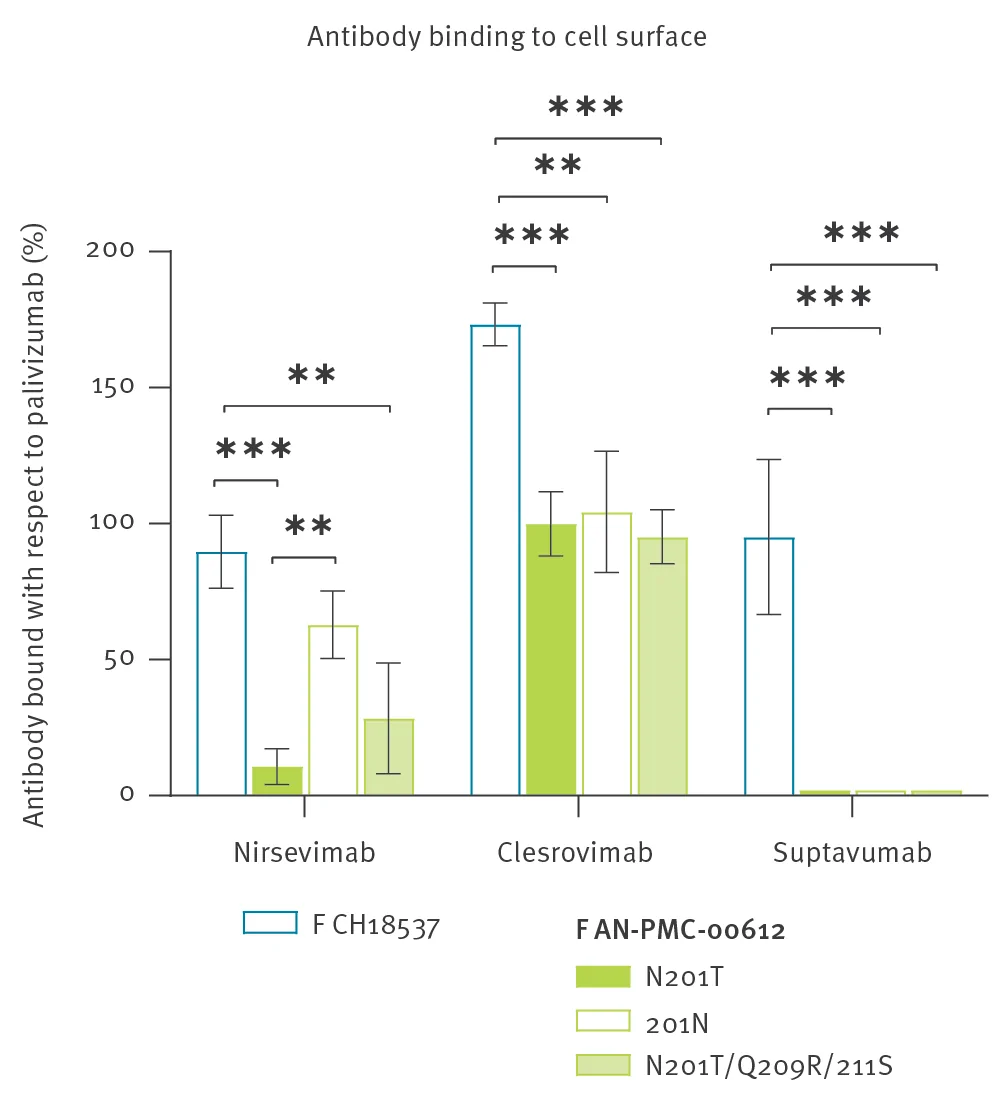
Effect of N201T on monoclonal antibody binding to contemporary RSV-B F proteins in transfected HEK293-F cells, Spain, 2026

RSV-B: respiratory syncytial virus subtype B.

Relative binding of nirsevimab, clesrovimab and suptavumab to full-length, cell-surface-expressed RSV F proteins was assessed by flow cytometry in transfected HEK293-F cells and normalised to palivizumab binding. Palivizumab, which targets antigenic site II, was used as a reference for protein expression because its binding is conformation-independent and no amino acid changes were detected at this site in the F proteins tested. The F proteins correspond to a historical RSV-B reference strain (blue open bar, F CH18537) and three constructs derived from the contemporary 2026 RSV-B F protein associated with Case 2 (hRSV/B/Spain/AN-PMC-00612/2026): the original F protein carrying F:N201T (green filled bar, F AN-PMC-00612 N201T); its N201 revertant (green open bar, F AN-PMC-00612 201N); and a derivative carrying F in the historical antigenic site Ø background (light green filled bar, F AN-PMC-00612 N201T/Q209R/211S). Presence of F:N201T reduced nirsevimab binding approximately 10-fold. Clesrovimab binding was moderately reduced in contemporary RSV-B F proteins, while remaining comparable to or higher than palivizumab binding. As expected, contemporary RSV-B F proteins showed markedly impaired suptavumab binding.

Data represent mean ± standard deviation (SD) (n = 3); *** p < 0.001, ** p < 0.01; Tukey’s Honestly Significant Difference test.

Clesrovimab binding was reduced in the contemporary strains but remained comparable to or higher than palivizumab binding. This reduction was maintained after reversion of N201T, suggesting that it is unrelated to this substitution and may reflect altered epitope exposure associated with other substitutions in contemporary RSV-B viruses. In contrast, suptavumab binding was markedly impaired in the contemporary RSV-B constructs, as expected from the naturally occurring L172Q and S173L substitutions associated with loss of suptavumab activity [4]. Reproduction of this established phenotype supports the capacity of the expression system to detect biologically relevant antigenic changes in circulating RSV-B strains.

## Discussion

Respiratory syncytial virus remains a major cause of acute lower respiratory tract infection in young children [5]. Long-acting monoclonal antibodies and maternal vaccines have substantially changed RSV prevention strategies [6], and the widespread implementation of nirsevimab in Spain since the 2023/24 season has been associated with a marked reduction in RSV-related hospitalisations [7]. Nirsevimab targets antigenic site Ø of the prefusion F protein [8]. Although substitutions affecting susceptibility remain uncommon in routine surveillance [9], F:N201T has previously been associated with reduced nirsevimab recognition in historical RSV-B backgrounds [10,11].

This report combines clinical surveillance, genomic analysis and functional antigenic characterisation of naturally occurring F:N201T-positive RSV-B viruses detected during widespread nirsevimab use. The case requiring PICU admission demonstrates that viruses carrying this substitution remain clinically competent and can be associated with severe lower respiratory tract disease. However, the small number of cases in this study do not allow estimation of clinical risk, breakthrough frequency or in vivo nirsevimab effectiveness against F:N201T-containing viruses.

The marked reduction in binding observed in contemporary F protein backgrounds indicates that additional substitutions around antigenic site Ø do not compensate for the antigenic effect of N201T. This is consistent with previous experimental evidence that some escape-associated substitutions within site Ø can reduce monoclonal antibody susceptibility while retaining viral replication capacity [12]. These findings support targeted investigation of F:N201T when detected in nirsevimab-immunised children, particularly in severe cases or epidemiological clusters.

Available genomic data currently support recurrent, sporadic emergence rather than sustained expansion. This pattern is compatible with convergent evolution, in which similar structural or antigenic constraints favour repeated acquisition of the same substitution in distinct viral backgrounds. It may partly resemble the diversification of antigenic site V that contributed to reduced population-level efficacy of suptavumab against RSV-B [4]. Although widespread nirsevimab use could increase opportunities for selection of variants with reduced susceptibility, the present data do not demonstrate selection-driven expansion or sustained transmission.

This study has several limitations. Only three recent cases were identified, infectious virus could not be recovered, and neutralisation assays using clinical isolates were not performed. Binding assays do not fully reproduce in vivo protection, and no causal relationship can be established between F:N201T, disease severity and nirsevimab failure. Publicly available genomic data are also affected by geographical and temporal differences in sampling intensity. Continued surveillance will be required to determine whether F:N201T detections remain sporadic or increase with broader use of antibody-based prevention.

## Conclusion

This study demonstrates that the RSV-B F:N201T substitution occurs in naturally circulating viruses, it markedly reduces nirsevimab binding in contemporary genetic backgrounds and has emerged repeatedly across distinct viral lineages. These findings support integrating genomic surveillance with functional antigenic characterisation to enable the early identification and assessment of RSV variants with reduced susceptibility to antibody-based prevention strategies.

## Data Availability

The eight sequences included in the genomic analysis, comprising the three sequences submitted by the participating hospitals and the additional sequences included to provide genomic context, are available through the GISAID Initiative as dataset EPI_SET_260902rq (https://doi.org/10.55876/gis8.260902rq).

## References

[1] Instituto de Salud Carlos III. Vigilancia centinela de Infección Respiratoria Aguda en Atención Primaria (IRAs) y en Hospitales (IRAG) Gripe, COVID-19 y VRS, temporada 2024-2025. España. [Sentinel surveillance of acute respiratory infection (ARI) in primary care and severe acute respiratory infection (SARI) in hospitals: influenza, COVID-19 and respiratory syncytial virus (RSV), 2024-2025 season. Spain.]. Madrid; Instituto de Salud Carlos III; 2025. Available from: https://cne.isciii.es/es/servicios/enfermedades-transmisibles/enfermedades-a-z/gripe-covid-19-y-otros-virus-respiratorios

[2] Shu Y McCauley J. GISAID: from vision to reality. Euro Surveill. 2017;22(13). 10.2807/1560-7917.ES.2017.22.13.30494 28382917 PMC5388101

[3] Gilman MS Moin SM Mas V Chen M Patel NK Kramer K Characterization of a prefusion-specific antibody that recognizes a quaternary, cleavage-dependent epitope on the RSV fusion glycoprotein. PLoS Pathog. 2015;11(7):e1005035. 10.1371/journal.ppat.1005035 26161532 PMC4498696

[4] Simões EAF Forleo-Neto E Geba GP Kamal M Yang F Cicirello H Suptavumab for the prevention of medically attended respiratory syncytial virus infection in preterm infants. Clin Infect Dis. 2021;73(11):e4400-8. 10.1093/cid/ciaa951 32897368 PMC8653633

[5] Li Y Wang X Blau DM Caballero MT Feikin DR Gill CJ Global, regional, and national disease burden estimates of acute lower respiratory infections due to respiratory syncytial virus in children younger than 5 years in 2019: a systematic analysis. Lancet. 2022;399(10340):2047-64. 10.1016/S0140-6736(22)00478-0 35598608 PMC7613574

[6] Mazur NI Terstappen J Baral R Bardají A Beutels P Buchholz UJ Respiratory syncytial virus prevention within reach: the vaccine and monoclonal antibody landscape. Lancet Infect Dis. 2023;23(1):e2-21. 10.1016/S1473-3099(22)00291-2 35952703 PMC9896921

[7] Núñez O Juaneda J Martinez-Marcos M Muñoz Platón E Rivas Wagner E Santiago-Pérez MI Two-season effectiveness of a single nirsevimab dose against RSV hospitalisation in healthy term-born infants: a population-based case-control study, Spain, October 2023 to March 2025. Euro Surveill. 2026;31(9):2500593. 10.2807/1560-7917.ES.2026.31.9.2500593 41788029 PMC13074286

[8] Zhu Q McLellan JS Kallewaard NL Ulbrandt ND Palaszynski S Zhang J A highly potent extended half-life antibody as a potential RSV vaccine surrogate for all infants. Sci Transl Med. 2017;9(388):eaaj1928. 10.1126/scitranslmed.aaj1928 28469033

[9] Fourati S Reslan A Bourret J Casalegno JS Rahou Y Chollet L Genotypic and phenotypic characterisation of respiratory syncytial virus after nirsevimab breakthrough infections: a large, multicentre, observational, real-world study. Lancet Infect Dis. 2025;25(3):301-11. 10.1016/S1473-3099(24)00570-X 39419046

[10] Simonich CAL McMahon TE Juviler G Kampman L Chu HY Bloom JD. Mutational constraints on RSV F and its neutralization by antibodies. bioRxiv. Preprint. 2026.02.12.705519; 10.64898/2026.02.12.705519

[11] Wilkins D Langedijk AC Lebbink RJ Morehouse C Abram ME Ahani B Nirsevimab binding-site conservation in respiratory syncytial virus fusion glycoprotein worldwide between 1956 and 2021: an analysis of observational study sequencing data. Lancet Infect Dis. 2023;23(7):856-66. 10.1016/S1473-3099(23)00062-2 36940703

[12] Zhu Q Lu B McTamney P Palaszynski S Diallo S Ren K Prevalence and significance of substitutions in the fusion protein of respiratory syncytial virus resulting in neutralization escape from antibody MEDI8897. J Infect Dis. 2018;218(4):572-80. 10.1093/infdis/jiy189 29617879

